# Distinctive Serum miRNA Profile in Mouse Models of Striated Muscular Pathologies

**DOI:** 10.1371/journal.pone.0055281

**Published:** 2013-02-13

**Authors:** Nicolas Vignier, Fatima Amor, Paul Fogel, Angélique Duvallet, Jérôme Poupiot, Sabine Charrier, Michel Arock, Marie Montus, Isabelle Nelson, Isabelle Richard, Lucie Carrier, Laurent Servais, Thomas Voit, Gisèle Bonne, David Israeli

**Affiliations:** 1 Inserm, UMRS_974, Paris, France; 2 Université Pierre et Marie Curie- Paris 6, UM 76, CNRS, UMR 7215, Institut de Myologie, IFR14, Paris, France; 3 Généthon, Evry, France; 4 Statistical consulting, Paris, France; 5 AP-HP, Groupe Hospitalier Pitié-Salpêtrière Charles-Foix, Unité Fonctionnelle d’Hématologie et d’Hémostase, Paris, France; 6 CNRS UMR8587, LAMBE, Evry, France; 7 Department of Experimental Pharmacology and Toxicology, Cardiovascular Research Center, University Medical Center Hamburg-Eppendorf, Hamburg, Germany; 8 Department of Therapeutic Trials and Databases, Institut de Myologie, Paris, France; 9 AP-HP, Groupe Hospitalier Pitié-Salpêtrière, U.F. Cardiogénétique et Myogénétique, Service de Biochimie Métabolique, Paris, France; The John Curtin School of Medical Research, Australia

## Abstract

Biomarkers are critically important for disease diagnosis and monitoring. In particular, close monitoring of disease evolution is eminently required for the evaluation of therapeutic treatments. Classical monitoring methods in muscular dystrophies are largely based on histological and molecular analyses of muscle biopsies. Such biopsies are invasive and therefore difficult to obtain. The serum protein creatine kinase is a useful biomarker, which is however not specific for a given pathology and correlates poorly with the severity or course of the muscular pathology. The aim of the present study was the systematic evaluation of serum microRNAs (miRNAs) as biomarkers in striated muscle pathologies. Mouse models for five striated muscle pathologies were investigated: Duchenne muscular dystrophy (DMD), limb-girdle muscular dystrophy type 2D (LGMD2D), limb-girdle muscular dystrophy type 2C (LGMD2C), Emery-Dreifuss muscular dystrophy (EDMD) and hypertrophic cardiomyopathy (HCM). Two-step RT-qPCR methodology was elaborated, using two different RT-qPCR miRNA quantification technologies. We identified miRNA modulation in the serum of all the five mouse models. The most highly dysregulated serum miRNAs were found to be commonly upregulated in DMD, LGMD2D and LGMD2C mouse models, which all exhibit massive destruction of striated muscle tissues. Some of these miRNAs were down rather than upregulated in the EDMD mice, a model without massive myofiber destruction. The dysregulated miRNAs identified in the HCM model were different, with the exception of one dysregulated miRNA common to all pathologies. Importantly, a specific and distinctive circulating miRNA profile was identified for each studied pathological mouse model. The differential expression of a few dysregulated miRNAs in the DMD mice was further evaluated in DMD patients, providing new candidates of circulating miRNA biomarkers for DMD.

## Introduction

Muscular dystrophies are a large heterogeneous group of over 30 different inherited disorders characterized by muscle wasting and weakness of variable distribution and severity, with or without heart defects, manifesting at any age from birth to senescence, and resulting in significant morbidity and disability [1]. The diseases are defined and classified according to their genetic cause as well as clinical and pathological manifestation, the distribution of predominant muscle weakness and the presence or not of other organ involvement. Cardiac involvement can range from conduction defects, hypertrophic or dilated cardiomyopathy to cardiac sudden death or heart failure [2]. Cardiomyopathies also occur without skeletal muscle involvement and present a wide clinical and genetic heterogeneity [3].

The principal analytical methods for muscular dystrophies diagnosis are based on muscle biopsy immunohistochemistry and Western blot analyses, serum content of muscle creatine kinase (CK), electromyography, electrocardiography and DNA mutation analysis [1]. Measurements of CK concentration released from damaged fibers into the serum are performed routinely in hospital analytical laboratories [4]. However the use of the CK test has certain disadvantages, including lack of disease specificity, lack of correlation between expression level and time course of the disease, and frequent false-positive results [4]. As for early cardiac disease diagnosis, serum markers such as NT-ProBNP might be reliable for early detection of myocardial dysfunction, but also lack specificity [5].

MicroRNA are short (22–24 nucleotides long) non-coding RNA that regulate mRNA post-transcriptionally either by promoting mRNA degradation or by attenuating protein translation [6]. Similar to mRNA and proteins, some miRNA species are expressed in a tissue-specific manner and regulated in different pathophysiological conditions [7], [8]. Therefore, monitoring the miRNA expression pattern permits the identification of the type and pathophysiological state of its tissue of origin. Some studies suggested that classification methods based on miRNA profiling are more accurate (specific and sensitive) compared to methods based on mRNA profiling [9], [10].

Specifically in striated muscles, the potential of using miRNA profile for classification of muscle pathologies has been elegantly demonstrated by Kunkel’s group, who identified distinct patterns of miRNAs in muscle biopsies derived from 10 major neuromuscular disorders [11]. MiRNA profiles were also reported for other muscle disorders [12] as well as in the context of heart failure [13]. However, the potential for use of this method for a practical clinical diagnosis application seems to be problematic. One major disadvantage is the invasive sampling of muscle or heart biopsies for miRNA extraction. Another important limitation is the variability of the miRNA expression profile between different striated muscles [14], rendering inconclusive any comparison between biopsies originating from non-identical muscles. Therefore, any comparative studies of miRNA expression patterns for analytical purposes in muscular dystrophies should be based on equivalent biological material from different patients.

Additional features have made miRNAs a particularly attractive research domain in the biomarker field. First, the molecular quantification methods for the analysis of miRNAs are relatively simple, fast and inexpensive. This is particularly important in comparison to the complex, expensive and time-consuming analytical methods used along the diagnostic process for analyzing expression of proteins and other biomolecules. Second, it has been discovered that miRNAs are secreted from the intracellular compartment into the extracellular environment, where they are stably expressed. Finally, it has been shown in human and in animal models that the circulating miRNA expression profile is dynamically changing in correlation with the pathophysiological state of the affected subjects [15], [16], [17], [18]. Therefore, profiling circulating miRNA can be indicative of pathophysiological status and these molecules may serve as biomarkers. This last point is crucial in multisystemic pathologies in which circulating miRNAs are conditioned by different affected tissues, integrating their tissue-specific effects. Thus, a comparative study of circulating miRNAs between affected subjects might be particularly appropriate for multi-systemic pathologies.

The field of circulating miRNA analysis for biomarker purposes is relatively new, including some significant publications from 2008 [15], [16], [17], [18]. These early proof-of-principle studies demonstrated the stability of miRNA expression in serum and plasma samples and focused on the development of the profiling technologies. Second phase studies focused on the discovery of circulating miRNA biomarkers in different pathologies, essentially in the fields of cancer and cardiovascular pathologies. They focused most often on identifying miRNA biomarkers specific for a given pathology, capable of indicating whether the biological sample was derived from an affected or a non-affected subject [19], [20].

In the present study we aimed at finding a combination of circulating miRNA biomarkers that could characterize, and discriminate different muscular pathologies. We selected five mouse models of striated muscle diseases. On the one hand, we have studied specificity of the circulating miRNA species in diseases that involve different organs and therefore different pathophysiological pathways, i.e. the skeletal muscles and/or the heart. On the other hand we have studied three closely related muscular dystrophies, thereby investigating and revealing the potential resolution of circulating miRNA analytical approach.

## Methods

### Ethical Declaration

The human study (DMD patients and controls) was conducted according the principles of the declaration of Helsinki “ethical principles for medical research”, and was specifically approved by the ethical committee CPP Ile de France VI, on July 20, 2010, and the Comité d’Ethique (412) du CHR La Citadelle (Liège, Belgium) on January 26^th^ 2011. Samples were collected from individuals under a written informed consent of parents or legal guardians.

The mouse strains included in this study are shown in Table 1. Mice were handled according to A1 biosafety requirements in accordance with the European guidelines for use of experimental animals (L358-86/609/EEC). All experiments were performed accordingly, to minimize animal discomfort. Prior to blood extraction mice were anesthetized by intraperitoneal injection of ketamine/xylazine. Anesthetized mice were sacrifice by cervical elongation at the end of the experiments.

**Table 1:** Principal features of mouse strains and models.

Pathology	Mutated gene/protein	Mouse model (abbreviationused thereafter)	Principal affected tissue	Reference
Limb-girdle muscular dystrophytype 2D, LGMD2D	*Sgca*/alpha-sarcoglycan	*Sgca*-null, (*Sgca*) backcrossed onC57BL/6J	Skeletal muscle	[24]
Limb-girdle muscular dystrophytype 2C, LGMD2C	*Sgcg*/gamma- sarcoglycan	*Sgcg*-null, (*Sgcg*) backcrossed onC57BL/6J	Skeletal muscle and heart	[25]
Duchenne muscular dystrophy,DMD	*Dmd*/dystrophin	*mdx* 4CV = *Dmd* null (*mdx*)backcrossed on C57BL/6J	Skeletal muscle and heart	[26]
Healthy control	None	C57BL/6		Jackson Laboratory
Emery-Dreifuss muscular dystrophy,EDMD	*Lmna*/lamin A/C	KI-*Lmna^p.H222P^ (Lmna* ki*)* 129/svJ/xC57BL/6	Skeletal muscle and heart	[31]
Healthy control	None	129/svJ/x C57BL/6 (*Lmna* wt)		
Hypertrophic cardiomyopathy,HCM	*Mybpc3*/cMyBP-C	KI-*Mybpc3^c.G815A^* (*Mybpc3* ki) 129/svJ × Blackswiss × CD1	Heart	[33]
Healthy control	None	129/svJ × Blackswiss × CD1 (*Mybpc3* wt)		

### Blood Collection

According the ethical requirements, human blood samples were collected from male subjects over 3 years-old and 15 kg in control as well as in genetically confirmed DMD patients. Peripheral blood samples were collected into 5 ml K3EDTA tubes (Greiner Bio-One). Plasma was separated from buffy coat and red blood cells after 10 minutes centrifugation 1800 g and stored at −80°C until further processing.

Mouse blood samples were either collected from the retro-orbital sinus or by intra-left ventricular puncture with 23 G needles, from anesthetized mice (ketamin 100 mg/Kg, xylazin 10 mg/Kg), into non-heparinized tubes in the absence of anti-coagulation treatment. Fresh blood samples were coagulated 30 min at room temperature and spun down at 10,000 g for 10 min. Supernatant was centrifuged again at 10,000 g for 10 min to separate serum from any residual cells and remaining cellular debris. Supernatant was collected into fresh tubes. For each strain, sera from 6 to 8 mice were pooled, divided in 3 aliquots for experimental triplicates, and stored at −80°C until further processing.

### Mouse Blood Count

One hundred µl of blood was collected from anesthetized mice by retro-orbital puncture using citrate 3.8% as anti-coagulant (1/10 Volume). Blood samples were analyzed for standard haematological parameters (white blood cell, red blood cells and platelet counts) using an MS9.3 counter (Schloessing Melet, Cergy-Pontoise, France).

### RNA Extraction and Quality Control Procedures

The miRVana PARIS (Ambion, Austin Texas) RNA extraction kit was identified in preliminary qualification experiment (data not shown) as suited for our experimental system.

Slightly different RNA extraction and quality control procedures were used in the two screening based on the two different technologies.

AB system: Total RNA was extracted from 625 µl of mouse serum. To perform extraction quality control, the synthetic miRNA cel-miR-39 (3.2 fentomole; Qiagen, France) was spiked in the crude mouse samples. RNA was eluted in 100 µl RNase-free water, precipitated overnight and resuspended in 10 µl RNase free water (AB). Total RNA was quantified by using a Nanodrop spectrophotometer (ND8000 Labtech, Wilmington Delaware) and analyzed using the Agilent small and pico RNA kit in the 2100 bioanalyzer (Agilent, Santa Clara California). RNA samples were systematically subjected to RT-qPCR quality control tests. This included stem-loop individual miRNA amplification reactions with primer for spiked-in cel-miR-39, the endogenous miRNA species miR-16, miR-142-3p, miR-223, miR-27a, miR-146b, and H_2_0 as a negative control.

Exiqon system: Total RNA was extracted from 300 µl of mouse serum. To perform quality controls, the synthetic small RNA Sp6 and Sp3 (10^8^ copies, Exiqon) were spiked in the crude mouse samples. RNA was eluted in 100 µl RNase-free water. Total RNA was quantified by using a Nanodrop spectrophotometer (ND8000 Labtech, Wilmington Delaware) and analyzed using the Agilent small and pico RNA kit in the 2100 bioanalyzer (Agilent, Santa Clara California). RNA samples were systematically subjected to RT-qPCR quality control tests. This included the use of spiked-in Sp3 (added before RNA extraction), Sp6 (added before RT reaction), the endogenous miRNA species miR-16, RNU5G (U5G small nuclear RNA) and H_2_0 as a negative control.

### RT-qPCR

miRNAs were quantified by both AB and Exiqon technologies. AB technology: total RNA (350 ng) was reverse-transcribed using the Megaplex™ Primers Pools A and B (rodents Version 2) and microRNAs were quantified with TaqMan® Array MicroRNA Cards A and B (rodents version 2) on the 7900 HT Real-Time PCR System (AB) following manufacturer’s guidelines. Quantification cycle (Cq) values were calculated with the SDS software v2.3 using automatic baseline with a threshold fixed at 0.2.

Exiqon technology: total RNA (200 ng) was converted into poly-A primed universal cDNA and microRNAs were quantified with miRNA-specific LNA™ primers on LightCycler® 480 Real Time PCR system (Roche Applied Science, Indianapolis Indiana) following manufacturer’s guidelines. Quantification cycle (Cq) values were calculated using the second derivative maximum algorithm.

### Data Processing and Statistical Methodology

MicroRNA expression RT-qPCR results, expressed as raw Cq with a threshold Cq ≤35, were normalized in the large-scale screenings to the calculated mean Cq of the sample [21]. Briefly: For each strain the pool of serum was divided in three aliquots. From each aliquot one cDNA was synthesized and each miRNA was PCR-amplified in triplicate from each cDNA. The average Cq of each PCR triplicate was calculated, thus giving three Cq values for each miRNA per strain. The mean Cq over the entire expressed miRNA (normalizer) was calculated for each strain. Delta Cq was calculated as the difference between each individual Cq and the normalizer. A corrected Cq was calculated as delta Cq+mean normalizer over all samples.

Individual miRNAs RT-qPCR assays were normalized relatively to the most stable identified referents miR-29a and miR-30b.

Differential expression was calculated using the 2^−ΔΔCt^ method, and miRNAs were considered differentially-expressed beyond a threshold of a 1.5 fold change (FC). Hierarchical clustering analysis (HC) [22], as well as Non-negative matrix factorization (NMF) analyses were performed with the “Array Studio” software package (OmicSoft Corporation, Cary North Carolina). All p-values were adjusted for test multiplicity using Benjamini-Hochberg False Discovery Rate method [23].

## Results

### Experimental Design

As we aimed to identify circulating miRNA biomarker profile of different muscular pathologies, we have selected 5 mouse models of skeletal and/or cardiac muscle diseases that involved different pathophysiological pathways and different degree of impairment of either tissue. A second objective of our study was to investigate the resolution power of circulating miRNA profile. To this end, we have compared the miRNA profile specificity of three mouse models of closely related muscular dystrophies.

The mouse strains included in the study are listed in Table 1. *Sgca*-null mice were used as models for LGMD2D [24], *Sgcg*-null mice for LGMD2C [25], *mdx*-4CV mice for DMD [26]. They result from mutations in the alpha-sarcoglycan (*Sgca*), gamma-sarcoglycan (*Sgcg*) and dystrophin (*Dmd*) genes, respectively, which all encode proteins of the dystrophin associated protein complex (DAPC). The DAPC connects the cytoskeleton of a muscle fiber to the surrounding extracellular matrix through the cell membrane. The absence of one of these proteins is associated with disruption of the DAPC and destabilizes muscle fibers [27]. Fiber degeneration in these muscular dystrophies is compensated by fiber regeneration process. Disease severity is progressing with ageing and in correlation with a slowdown of this compensatory process. These DAPC-associated myopathies affect skeletal muscles but are also associated with cardiac dilation to a variable degree, ranging from only rare and mild symptoms in LGMD2D patients [28] and mouse model [29] to more pronounced cardiac pathology in LGMD2C and DMD patients and mouse models [30]. The three mouse models for the DAPC-associated pathologies are inbred strains, bred on the genetic background of the C57BL/6J mouse, which was included in the study as their wild type control strain.

The two other strains were models for Emery-Dreifuss muscular dystrophy (EDMD) and hypertrophic cardiomyopathy (HCM). The EDMD mouse model is a knock-in (KI-*Lmna^p.H222P^*) that reproduces the p.H222P lamin A/C substitution identified in EDMD patients [31]. Lamins A and C are ubiquitously expressed nuclear envelope proteins both encoded by the *LMNA* gene. *LMNA* mutations were reported in more than 10 distinct pathologies [32]. The homozygous mice develop, similarly to human patients, progressive muscular dystrophy associated with dilated cardiomyopathy [31]. This model has a specific mixed genetic background of 129/svJ and C57BL/6 and wild-type littermates were used as controls. Lastly, the HCM mouse model corresponds to the knock-in of human *Mybpc3* mutation (KI-*Mybpc3^c.G815A^*) [33] which is frequently associated with HCM in human [34]. *Mybpc3* codes for cardiac myosin binding protein C (cMyBP-C) exclusively expressed in both human and mouse cardiomyocytes [35]. Consequently the cMyBP-C related HCM is cardiac specific. This model also has a specific mixed genetic background of 129/svJ and Blackswiss/CD1 and wild-type littermates were used as controls. All studied mice were 9–11 week-old, which are clinically relevant ages in all pathological models studied [20]–[22], [24], [26].

### Screening Strategy

We compared miRNAs expression levels in mice sera pools, collected from 6 to 8 mice per pool. In a preliminary experiment, we have compared the serum miRNA expression levels between biological and technical triplicates serum pools. We have found that the standard deviations were essentially the same in the two approaches (data not shown), showing that variation in miRNA expression levels between triplicates is almost exclusively technical rather than biological, thus validating a choice of technical triplicates pools screening.

**Figure 1:**
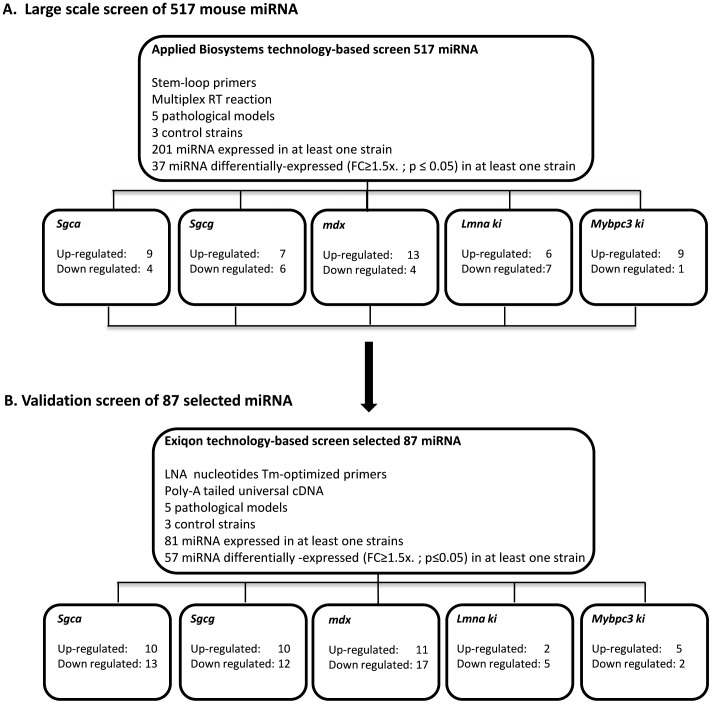
Screening strategy. Serum miRNAs were analyzed in 5 mouse pathological models, the *Sgca*-null *(Sgca)* the *Sgcg*-null *(Sgcg)*, the *mdx-*4CV *(mdx)*, the KI-*Lmna^p.H222P^* (*Lmna* ki) and the KI-*Mybpc3^c.G815A^* (*MyBpc3* ki) and their respective 3 healthy controls. For each mouse strain, sera were pooled from 6 to 8 mice at the age of 10 weeks. A first step large-scale screening of 517 mouse miRNAs was performed with the AB TaqMan® Array Rodent MicroRNA A and B Cards (Set v2.0). Out of 201 miRNAs detected (Cq ≤35 in at least one strain), 37 were differentially expressed (FC≥1.5x, p≤0.05) in at least one pathological strain. Numbers of dysregulated miRNAs per strain are indicated in the figure. A restricted list of 87 miRNAs, was selected for a second-step validation screening, performed with the Exiqon miRCURY LNA™ Universal RT microRNA PCR. Out of 81 detected miRNAs, 57 were differentially expressed (FC≥1.5x, p≤0.05) in at least one pathological model. The numbers of dysregulated miRNAs per strain are indicated in the boxes at the lower part of the figure and correspond to the double positive dysregulated miRNAs, FC≥1.5x in both screenings, and p≤0.05 in the second screen.

To obtain maximum miRNA detection sensitivity and specificity, we adopted a two-step RT-qPCR approach (Figure 1). First a large-scale miRnome screening was performed with the TaqMan® Array MicroRNA Cards A and B (rodent genome-wide miRNA, miRBase 16 annotations) testing 517 mouse miRNAs. Out of 201 miRNAs detected (Cq≤35 in at least one strain), 37 were differentially expressed (FC≥1.5x, p≤0.05) in at least one pathological strain. Second, for further validation studies we took advantage of an alternative RT-qPCR technology. We used the Pick-&-Mix microRNA PCR Panels for large screening and microRNA LNA™ PCR primer sets for individual assays, both provided by Exiqon. In this second step, we utilized a limited rather than a full scale miRNA database screen. We selected miRNAs that were identified in the first screening, i.e. the 37 differentially expressed, and some additional differentially expressed miRNAs with smaller FC value, as well as a collection of miRNAs that are known to be expressed in normal and pathological muscle, for a total of 87 miRNA. (Table S1). Raw data results of this screening are shown in table S2.

### Pathological Models Clustered Separately

**Figure 2:**
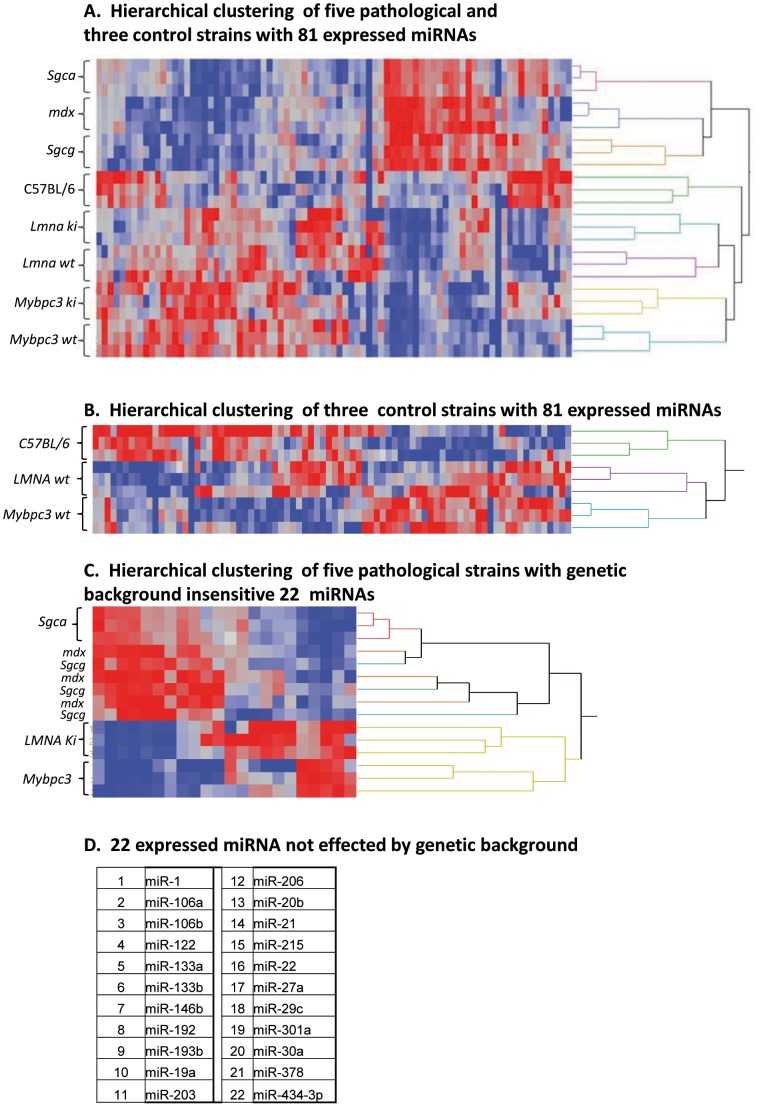
Hierarchical clustering of 81 serum miRNAs expression level in mouse models for muscular diseases. The pathological models included in the study are the *Sgca*-null *(Sgca)* the *Sgcg*-null *(Sgcg)*, the *mdx-*4CV *(mdx)*, the wild type C57BL/6 healthy control mice; the KI-*Lmna^p.H222P^* (*Lmna* ki) and its healthy littermate control (*Lmna* wt) mice and the KI-*Mybpc3^c.G815A^* (*MyBpc3* ki) and its healthy control (*MyBpc3* wt) mice. Total RNA was extracted from serum samples and a two-step miRNA quantification strategy was employed as detailed in Figure 1. (A) Valid expression data of 81 miRNAs were subjected to a hierarchical clustering analysis, performed with the Array Studio software. (B) Hierarchical clustering analysis of the three healthy control strains with the expression data of 81 miRNA. (C) Hierarchical clustering analysis of the 5 pathological models strains with the expression data of 22 expressed miRNAs minimally affected by genetic background, listed in (D). Expression level of the miRNAs is presented in color-code with gradient between the dark-blue, representing the lowest expression level, to the deep-read for the highest expression level.

Eighty-one miRNAs from the 87 assays were detected (as expressed below Cq≤35) in the second step validation screen (Figure 1). Remarkably, the hierarchical clustering (HC) analysis of the miRNAs expression data demonstrated perfect triplicate clustering and strain separation pattern in all the mouse models (Figure 2a), with a sub-grouping segregation into three principal clusters; the first was composed of the DAPC-associated myopathies and their C57BL/6 control mice, the second and third clusters were composed of the two other pathologic mouse models, each one with its control strain. This observed sub-grouping segregation into three principal clustered correlated not only with three distinctive types of pathologies but also with the three different genetic backgrounds that participated in our survey. Indeed HC analysis of triplicate samples of the three healthy control mice strains demonstrated again perfect clustering (Figure 2b). A non-negative matrix factorization (NMF) analysis was employed in order to identify miRNAs that were minimally participated in the genetic background clustering pattern, allowing the identification of 22 such miRNAs (Figure 2d). HC analysis of the 5 pathological strains with these 22 expressed miRNAs allowed almost perfect triplicates clustering; with however mix between the samples derived from the *Sgcg*-null and the *mdx* mouse models (Figure 2c).

### Evidence for Dysregulated miRNAs in the Pathological Models

In this study, dysregulated miRNA was defined as up or downregulated miRNA by at least 1.5× FC in both screens (double positive) and a p value ≤0.05 in the second (Exiqon) screen, excepted for two borderline miRNAs of particular interest (1.5<FC<1.4). A complete list of the dysregulated serum miRNAs is presented in Table 2. The largest number of 28 dysregulated miRNAs was found in the DMD model, and the smallest one of 7 dysregulated miRNAs was found in both EDMD and the HCM models. In the three DAPC-associated myopathy models characterized by massive tissue destruction, the maximal FC of upregulated miRNAs largely exceeded the maximal FC of downregulated miRNAs. Maximal upregulated and downregulated FC was more balanced in the models of the two other pathologies, which are characterized by only moderate tissue destruction [12], [32], [35]. In agreement with a recent publication [36] we identified strong activation in dystrophic mice of the muscle-enriched miR-1, miR-133a, miR-133b and miR-206. This activation was not specific to the *mdx* mouse; strong activation of these 4 miRNAs was identified in all three DAPC-associated myopathies (Tables 2 and 3). In addition to this previously described activation in the serum of muscle-enriched miRNA species, we detected the deregulation in the serum of other miRNA species, many of them common to the three DAPC-associated myopathy models. This included the markedly upregulated miR-378, which is another muscle-enriched miRNA [37] as well as miR-193b, miR-149 and miR-30a. The most repressed miRNA in all three DAPC-associated myopathy models was miR-122. Another notable common downregulated miRNA in the DAPC-associated pathologies was miR-31, that is known to regulate the expression of the dystrophin gene [38].

**Table 2:** Differentially expressed miRNAs by strain.

Dysregulated miRNAs (FC ≥1.5 x)																			
*Sgca*-null mice				*Sgcg*-null mice				*mdx* 4CV mice				KI-*Lmna^p.H222P^* mice				KI-*Mybpc3^c.A815G^* mice			
Upregulated		Downregulated		Upregulated		Downregulated		Upregulated		Downregulated		Upregulated		Downregulated		Upregulated		Downregulated	
miRNA id	FC	miRNA id	FC	miRNA id	FC	miRNA id	FC	miRNA id	FC	miRNA id	FC	miRNA id	FC	miRNA id	FC	miRNA id	FC	miRNA id	FC
miR-206	23.64	miR-122	−3.8	miR-133b	24.3	miR-125a-5p	−2.78	miR-206	36.68	miR-122	−4.94	miR-146b	2.24	miR-130a	−1.92	miR-192	1.87	miR-451	−5.2
miR-133b	15.92	miR-672	−2.19	miR-133a	19.19	miR-31	−3.09	miR-133b	27.17	miR-429	−3.81	miR-200a	1.62	miR-133a	−1.88	miR-429	1.63	miR-301a	−**2.38**
miR-1	14.14	miR-125a-5p	−**2.02**	miR-206	12.99	miR-26b	−2.55	miR-133a	20.52	miR-200a	−3.69			miR-133b	−1.82	miR-125b-5p	1.55	let-7i	−2.1
miR-133a	12.11	miR-200a	−1.98	miR-1	9.86	miR-142-3p	−2.19	miR-1	10.32	miR-672	−3.49			miR-1	−**1.73**	miR-187	1.52		
miR-378	3.91	miR-199a-3p	−1.95	miR-378	6.82	miR-429	−2.13	miR-378	7.43	miR-31	−2.42			miR-151-3p	−1.71	miR-200a	1,48		
miR-193b	2.23	miR-195	−1.88	miR-30d	4.8	miR-26a	−**2.12**	miR-193b	4.47	miR-451	−2.33			miR-339-3p	−1.68				
miR-149	2.2	miR-429	−1.83	miR-193b	3.72	miR-200a	−2.12	miR-30d	3.81	miR-143	−2.24								
miR-30a	2.16	miR-151-3p	−1.76	miR-22	3.48	miR-122	−2.12	miR-149	2.98	miR-195	−2.19								
miR-30d	**1.92**	miR-31	−1.75	miR-149	2.81	miR-672	−2.01	miR-30a	2.84	miR-148a	−2.12								
miR-709	1.68	miR-26a	−**1.72**	miR-30a	2.72	let-7g	−1.94	miR-434-3p	2.38	let-7g	−2.11								
miR-30e	1.61	miR-125b-5p	−1.71	miR-106a	**1.7**	miR-125b-5p	−1.92	miR-146b	**1.89**	miR-125b-5p	−2.09								
		miR-142-3p	−1.7			let-7b	−**1.83**	miR-30e	1.74	miR-200b	−2.03								
		miR-152	−1.6			let-7i	−1.72			miR-145	−2.02								
		miR-301b	−1.,6			miR-215	−**1.67**			miR-142-3p	−2.02								
		miR-93*	−1.58			miR-301b	−1.53			let-7b	−**1.88**								
		miR-200b	−**1.56**							miR-26b	−**1.77**								
										miR-152	−1.75								
										let-7i	−1.6								
										miR-301b	−1.41								

Dysregulated miRNAs in all mouse models. Positive or negative FC values indicated up or downregulated miRNA respectively compared to control. Listed miRNAs which are “double positive”, i.e. are dysregulated at least 1.5× FC in both AB and Exiqon technology-based screens (except miR-301b in *mdx* and miR 200a in *Mybpc3* ki with FC slightly below 1.5). The FC and p values were derived from the Exiqon-based screening. FC values are significant (p≤0.05), except those marked in red.

**Table 3:** Commonly dysregulated miRNAs in all mouse models.

Dysregulated miRNAs common in DAPC-associated myopathy models and serum creatine kinase level (last line) (up and downregulated) Fold change value					Expression in other models	
No.	miRNA ID	*Sgca*-null mice	*mdx* 4CV mice	*Sgcg*-null mice	*KI-Lmna^p.H222P^* EDMD mice	*KI-Mybpc3^c.A815G^* HCM mice
1	mmu-miR-206	23.64	36.68	12.99		
2	mmu-miR-133b	15.92	27.17	24.30	−1.88	
3	mmu-miR-133a	12.11	20.52	19.19	−1.82	
4	mmu-miR-1	14.14	10.32	9.86	−1.73	
5	mmu-miR-378	3.91	7.43	6.82		
6	mmu-miR-193b	2.23	4.47	3.72		
7	mmu-miR-149	2.20	2.98	2.81		
8	mmu-miR-30a	2.16	2.84	2.72		
9	mmu-miR-301b	−1.60	−1.41	−1.53		
10	mmu-miR-142-3p	−1.70	−2.02	−2.19		
11	mmu-miR-31	−1.75	−2.42	−3.09		
12	mmu-miR-672	−2.19	−3.49	−2.01		
13	mmu-miR-200a	−1.98	−3.69	−2.12	1.62	1.48
14	mmu-miR-429	−1.83	−3.81	−2.13		
15	mmu-miR-122	−3.80	−4.94	−2.12		
	Creatine kinase	17.18	8.63	29.6	−3.44	

All miRNA species listed are “double positive”, i.e. are dysregulated at least 1.5× FC in both AB and Exiqon technology-based screenings (except miR-200a in KI-*Mybpc3^c.A815G^*, FC = 1.48). FC values are derived from the Exiqon-based screening. The order is of descending FC values in the *mdx* model. All p values are significant (p≤0.05, Exiqon screen), except for the miR-1 in the KI-*Lmna^p.H222P^* mouse.

The miR-133a, miR-133b, miR-1, strongly upregulated in the DAPC-associated myopathy models, were slightly but significantly downregulated in the EDMD mouse model. In correlation, the serum CK level (Table 3, bottom line) was upregulated in the three degenerative DAPC-associated myopathy models and downregulated in the EDMD mouse.

The only common dysregulated miRNA in all models studied, miR-200a, was downregulated in the DAPC-associated pathologies and upregulated in the two other pathologies.

**Figure 3:**
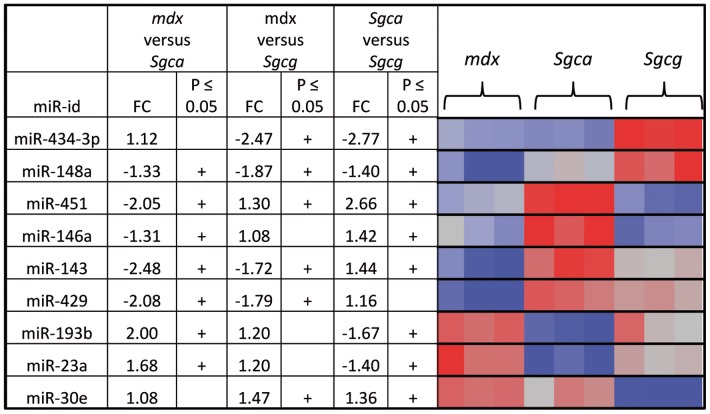
Specificity of serum miRNA profile in three DAPC-associated mouse models. NMF analysis of miRNA expression levels of 9 serum miRNAs in *Sgca*-null (*Sgca*), *Sgcg*-null (*Sgcg*), and *mdx*-4CV mice (*mdx*). Serum samples were collected and miRNA expression analyses were done as detailed in Figure 1. One experiment out of two performed is presented. (+) significant p value (p≤0.05).

The three DAPC-associated myopathy mouse models studied here have the same genetic background and were compared consequently to the same control mouse strain (C57BL/6J). These three pathologies shared many dysregulated miRNAs and presented consequently very similar serum miRNA signatures. Relatively to their common C57BL/6J controls, they did not exhibit pathology-specific dysregulated miRNA patterns. For example, miR-22 that was upregulated in the *Sgcg*-null mouse in the Exiqon screen (FC = 3.48, p≤0.05, table 2), was also significantly upregulated in the same screen in the *Sgca*-null and the *mdx* mouse models. However in these two latter models miR-22 was not upregulated in the AB screening and therefore was not a double positive candidate and absent from table 2 for the *Sgca*-null and the *mdx* models. However the HC analysis (Figure 2a) clustered these 3 pathologies separately, indicating a distinct and specific serum miRNA signature for each one. We therefore compared these three pathologies directly with each other (instead of their common control strain). Indeed the direct comparison of the expressed miRNAs in the LGMD2D, LGMD2C and the DMD mouse models identified 9 differentially expressed miRNAs which provided a disease-specific signature for these three pathologies (Figure 3).

Taken together, the double screening approach used in this study allowed obtaining a perfect pathological model clustering pattern (Figure 2), composed of lists of dysregulated miRNAs that distinguish between unrelated striated muscle pathologies (Table 2), and a set of miRNAs that distinguishes between closely related muscular dystrophies (Figure 3).

### Blood Component Differences between Pathological Mouse Models

It has been suggested that an important source of serum miRNA is the hematopoietic system, and that blood components such as erythrocytes, platelets and leucocytes are contributing to the repertoire of circulating miRNAs [39]. We asked therefore whether blood counts were different between the pathological models. Indeed we found significantly (p<0.05) elevated level of leucocytes and platelets, but not erythrocytes, in all the three DAPC-associated myopathy mice compared to their common healthy control C57BL/6 mice (Figure S1). These results suggest a possible contribution of the hematopoietic system to the specific repertoire (profile) of the serum miRNAs identified in the distinct pathologies.

### Age-dependent miRNAs Dysregulation in the *mdx* Mice

**Figure 4:**
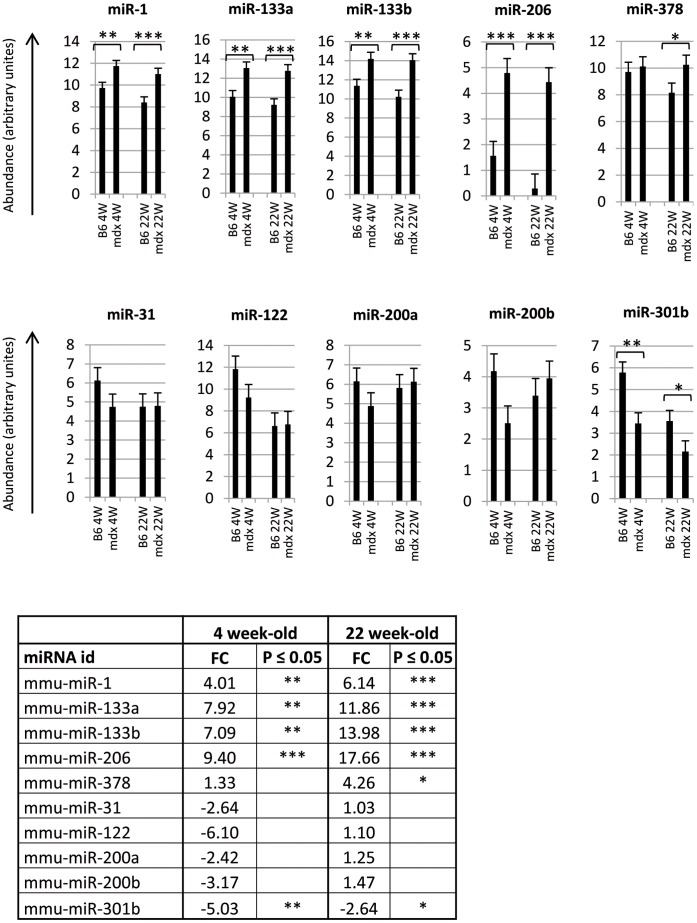
Serum miRNAs expression and fold change in *mdx*-4CV and control mouse at the ages of 4 and 22 weeks old. Expression of 10 miRNAs were studied in the serum of the *mdx*-4CV mouse at the ages of 4 weeks (mdx4W) and 22 weeks (mdx22W) and in age matched control C57BL/6 mice (B64W and B622W). Serum samples from 6 mice of each strain and time point were pooled and subjected to RT-qPCR quantification using Exiqon technology. Results are the average of three independent experiments. miRNA expression level, designated “abundance” on the vertical axis, is the log_2_ of the relative miRNA expression level normalized to miR-29a and 30b, found previously to be the most stable miRNAs in our experimental setup (data not shown). Standard deviation and p values are shown with *stands for p≤0.05, **stands for p≤0.01, and ***stands for p≤0.001. Values in the table are of the corresponding fold change (FC) values for the same miRNAs of the graphical presentation.

Serum expression levels of 5 upregulated and 5 downregulated miRNAs were further evaluated in the *mdx* mice at the ages of 4 and 22 weeks (Figure 4). In agreement with previous results from 10 week old mice (Table 2) we observed a marked upregulation of miR-1, miR-133a, miR-133b and miR-206 in the sera of *mdx* mice at ages of both 4 and 22 weeks. In contrast, miR-378 was activated significantly at 22-weeks of age, but not at 4 weeks of age. For miRNAs downregulated in 10-week-old *mdx* mice (Table 2), we have consistently observed downregulation at the age of 4 weeks, which however was statistically significant only for miR-301b. MiR-301b was still downregulated at the age of 22 weeks. Collectively, these results suggest that markedly activated serum miRNA species in the *mdx* mouse are upregulated over wide range of ages, while other more subtle changes in serum miRNA expression might be dysregulated in age-dependent manner.

### Dysregulated Plasma miRNAs in DMD Patients

**Figure 5:**
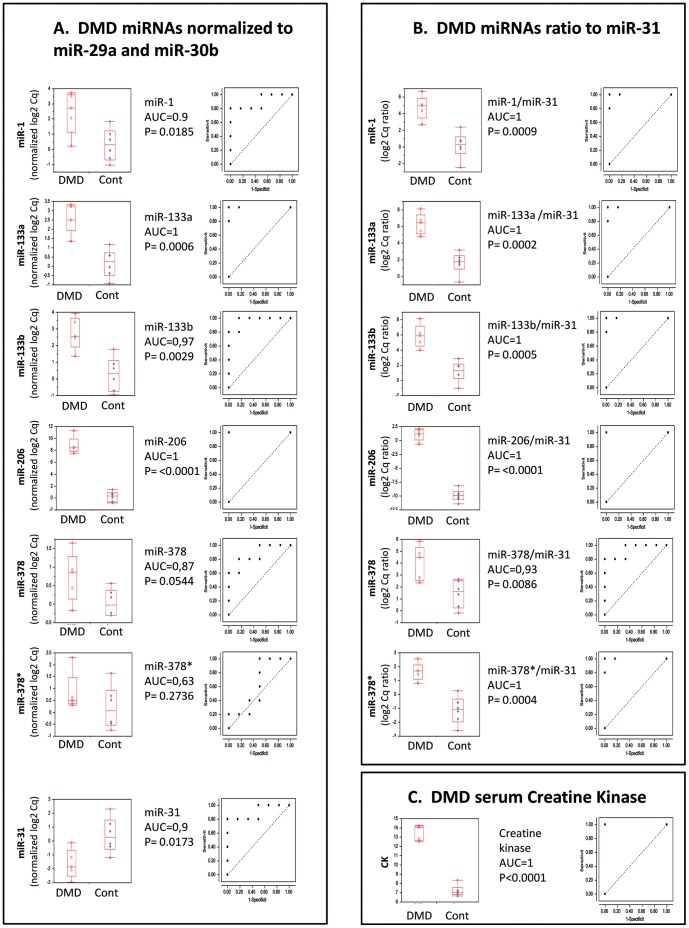
Dysregulated plasma miRNA in DMD patients. Expression data of 7 miRNAs were studied in a DMD cohort including 5 patients and age-matched controls (Table S3). (A) miRNA expression was normalized to miR-29a and miR-30b, with their respective ROC curves. (B), miRNA expression is presented relative to the miR-31 expression level of each patient, with their respective ROC curves. Individual miRNAs “area under the curve” (AUC) and p values are indicated respectively. (C) DMD plasma creatine kinase and its respective ROC curve. The vertical axe indicates the ratio of the log2 quantities of the tested miRNA relative to the normalizer miRNAs.

The principal circulating miRNAs identified in the *mdx* mouse were studied in DMD patients and their age matched controls (n = 5, table S3). In agreement with our *mdx* data, this analysis confirmed the upregulation of miR-1, miR-133a, miR-133b and miR-206, previously reported in DMD patients [36] and dystrophic dogs [40]. Additionally the upregulation of miR-378 and its co-transcribed partner miR-378* were confirmed. In contrast, the upregulation of miR-149 and miR-193b, and the downregulation of miR-122 and miR-200a were not confirmed in DMD patients (not shown). In agreement with the results of *mdx* mice, we observed differential expression of miR-31, which is to our knowledge the first downregulated circulating miRNA to be reported in plasma from DMD patients. MiRNA RT-qPCR raw data from DMD patients were either normalized to the stable circulating miRNAs miR-29a and 30b (Figure 5a), or alternatively presented as miRNA ratio to the downregulated miR-31 (Figure 5b), with their respective ROC curves, and with the CK measurements (Figure 5c).

## Discussion

The main goal of this study was the simultaneous evaluation of several disease models for their miRNA profile in order to identify specific sets of circulating miRNA biomarkers for each disease. The experimental approach adopted included the use of mouse models for a range of striated muscular diseases and the elaboration of a screening strategy composed of two successive steps of RT-qPCR miRNA profiling. The first step was a complete miRnome screening, while the second was a validation for a subset of candidates identified in the first one. Independent serum samples and different RT-qPCR technologies were used in the second step to reduce false positive detection of dysregulated miRNA.

Several miRNA species were dysregulated in 3 pathological models and in two screening systems in each model, thus in 6 independent screenings experiments. Therefore, it is likely that this strategy of genome-wide two-steps and two technological platforms screening of five pathological models in parallel contributed to the production of high quality data, interpreted by a perfect clustering pattern (Figure 2), and a reliable list of dysregulated miRNAs (Table 2). It should be noted however that defining as validated dysregulated miRNAs only the double positive candidates might have resulted in neglecting some truly dysregulated miRNAs. For example, a miRNA detected as dysregulated in one screen and failed to be detected as dysregulated in the other screen, or a miRNA detected as upregulated in the first screen and downregulated in the second screen. The latter example might be the case of miR-106b, which was upregulated 4.51x and 2.2x in the LGMD2D and DMD models respectively, in the AB screening, but downregulated −1.21x and −1.9x respectively in the same models in the Exiqon screening.

### Specificity of miRNA Profile to Pathology

A second major finding of this study was the identification of a disease-specific circulating miRNA signature for all five mouse models. Of note, two levels of specificity of miRNA signatures were identified. The first, in comparison to the control healthy mice, was able to detect pathologies of different categories. For example, the spectrum of dysregulated miRNAs in the DMD mouse model varied considerably from that of the EDMD mouse model. However the discrimination between the three muscular dystrophies, LGMD2D, LGMD2C and DMD failed because they shared the same dysregulated miRNA expression pattern as their common healthy control mouse C57BL/6. The second level of specificity of miRNA signature was achieved by direct comparison of the miRNA profiles of these three pathological models. Together, this provided circulating miRNA-specific profile for these three closely related pathological models as well. Closest miRNA profiles are those of the *mdx* and the *Sgcg*-null mice (Figure 2c) both affecting skeletal and cardiac muscles, while *Sgca*-null mouse affecting only skeletal muscles clustered separately.

### Circulating miRNA Profiling may Reflect Pathophysiological Changes over Variety of Tissues

Among the advantage of the serum biomarker approach is the ability of these biomarkers to reflect pathophysiological modifications in a multisystem genetic disorder not only in the primary affected tissue, muscles in the present study, but also in secondary affected tissues. In this context, the observation made here that the blood counts are significantly distinct between the pathological models and their respective controls is important. Indeed the DAPC-associated myopathies studied here are inflammatory pathologies, which might explain differences in the blood counts. Thus, these hematological differences might contribute to the differentially expressed serum miRNAs, increasing thus the overall analytical power of this method.

### Circulating Biomarker Profiling is Particularly useful in Muscular Dystrophies

The common dysregulated serum miRNAs in the DAPC-associated pathologies (Table 3) included the four principal muscle enriched miRNAs, miR-1, miR-133a, miR-133b and miR-206 [41], [42]. Together with miR-378, another myofiber-enriched miRNA [37], we identified these miRNAs as the most upregulated in the serum of mice of the DAPC-associated pathologies, far beyond the FC level of the most downregulated miRNA. Fold change values of circulating miRNAs in many pathologies and pathological models are often modest. Taking into account problems of technical variations and lack of precise normalization procedure, low FC values are of concern, as they may lead to false interpretation. The particular elevated miRNA FC values observed in muscular dystrophies suggest a particular analytical robustness in applying this method to this type of pathologies.

### Contradicting Trends between Intra- and Extra-cellular Dysregulated Muscle miRNAs

Massive contribution of tissue destruction to the circulating miRNA repertoire may explain another seemingly paradox. Downregulation of certain miRNAs, including miRNA-1, miRNA-133a and miRNA-133b in dystrophic muscle ([43] and our unpublished data) coincide with their upregulation in the serum. This might at first sight looks as contradicting the suggestion that concentration of extracellular miRNA is proportional to its intracellular concentration [44]. The level of some myofiber-enriched miRNAs may decrease in dystrophic compared to normal muscle biopsies as result of a switch in the transcriptional program in the dystrophic muscle and of a gradual replacement of contractile tissue by fibrotic and fatty tissues. Yet, ongoing muscle tissue destruction or cellular membrane leakage keep contributing muscle-originating miRNAs to the circulation despite their reduced intracellular concentration, thus reduced muscular concentration of some miRNA species in dystrophic mouse muscles coincides with their increased serum concentration. The downregulation of miR-31 in the serum is at present more difficult to interpret. Intramuscularly, miR-31 has been found strongly upregulated in dystrophic muscle, where it downregulates the expression of dystrophin [38]. In dystrophic subjects miR-1, miR-133a and miR-133b are downregulated in the muscle and upregulated in the serum while conversely miR-31 is upregulated in the muscle and downregulated in the serum.

### Dysregulated miRNAs in the non DAPC-associated Muscular Pathologies

Three out of the eight dysregulated miRNAs in the EDMD mouse model, miR-1, miR-133a and miR-133b, were found to be dysregulated in the DAPC-associated pathologies (Table 3), but with opposite trends. Thus, these myofibers-enriched miRNAs are upregulated in the serum in myopathies associated with massive muscle fibers destruction but downregulated in the serum of myopathies associated with low level of degenerative/regenerative process. In a nice correlation CK level was also downregulated in the EDMD serum. Indeed in the EDMD model the dystrophic process is milder than those observed in DAPC related myopathy models [24]. The pathomechanisms of *Lmna* mutations are thought to be related to increased mechanical stress sensitivity and/or abnormal signaling pathway/gene regulation in link with abnormal nuclear envelop structure and function [12], [33], [45]. The eight dysregulated miRNAs in the serum of the EDMD mouse model were not part of the previously reported dysregulated miRNAs detected in muscle biopsies of EDMD patients [12]. In EDMD, in absence of major fiber disruption or membrane leakage, the dysregulated miRNA species present in the circulation and in the muscle might not be closely related and may reflect complex pathophysiological mechanisms that deserve to be further explored.

The miR-200a was the only commonly dysregulated in *Mybpc3*-related HCM with the other 4 pathologies. The pathophysiology of the EDMD model and of the cMyBP-C related HCM models are very different one from the other and from the physiopathology of the DAPC-associated myopathy models. However, the main affected tissues are the skeletal muscles in the models for the DAPC-associated myopathies and for EDMD. In contrast, in the cMyBP-C related HCM model only the heart is affected. It was thus not surprising that more dysregulated circulating miRNAs were found in common to those pathologies that affect principally skeletal muscles than with the cMyBP-C related HCM model that affects the heart only.

The highest dysregulated miRNAs identified in the cMyBP-C related HCM model were the miR-192 and miR-429 for the upregulated, and the miR-451 and miR-301a for the downregulated. The study of circulating miRNA biomarkers for cardiovascular pathologies is a very active research domain. It is thus interesting to note that the cMyBP-C related HCM miRNAs biomarkers identified in the present study (see table 2 for a full list), are different from many recently identified miRNAs biomarkers for a range of cardiovascular ischemic pathologies [19], [46]. However the majority of the published circulating miRNA studies of cardiovascular pathologies and pathological models were focused on ischemic cardiomyopathies [19], [46], whereas the cMyBP-C related cardiomyopathy is not an ischemic heart disease, thus explaining a different dysregulated circulating miRNAs species.

### Age-dependent miRNA Dysregulation in mdx

The sub-selection of miRNAs studied in 4 and 22 week-old *mdx* mouse has provided additional information about the age-dependent evolution of these dysregulated miRNAs in muscular dystrophy. As expected, we noticed that while the expression of some dysregulated miRNAs was different from controls at all ages studied, the deregulation of others was age-dependent. In particular high FC values in the three ages studied were found for the muscle enriched miRNAs miR-1, 133a, 133b, and 206. It will be interesting to study these miRNAs in the DAPC-associated myopathies in older mice once myofiber degeneration is slowing down.

### Validation Studies in DMD Patients

Two recent studies reported the upregulation of miR-1, miR-133a, miR-133b and miR-206 in the serum of dystrophic animal models and human patients [36], [40]. Our experimentation in the *mdx* mice confirmed these previous reports and provided some new candidates. The small DMD cohort studied here support further the relevance of these previously identified biomarkers as well as the newly identified miR-378, miR-378* and miR-31. Of note is the downregulation of miR-31. This downregulation was observed in all three DAPC-associated pathologies in mice at the age of 10 weeks (Table 3) but was age dependent in the *mdx*, expressed to a similar level at the age of 22 weeks compare to the wild type (Figure 4). Its downregulation in the plasma in the DMD cohort is of particular interest since this is the first reported circulating downregulated miRNA in DMD patients.

In the standard ROC analysis (Figure 5a), the different circulating biomarkers are analyzed and classified after their normalization relative to the expression of stable miRNAs, mirR-29a and miR-30b. The downregulation of miR-31 led us to evaluate in DMD patients by ROC analysis, the ratio of the expression of the upregulated miRNAs to miR-31 (Figure 5b). The “ratio-to-miR-31” method reveal clear advantage over the standard normalization to the most stable miRNAs, by providing improved AUC and reduced p values in the ROC analysis. Additional advantage of the ratio method is that miR ratio remains stable over a wide range of RNA mass input, thus ratio comparison between samples is insensitive to variations in the input quantity of the analyzed RNA. A definitive approval of these proposed miRNAs, (and combination of miRNAs) biomarkers will have to wait for confirmation studies with larger DMD cohorts.

Compared to the study of Cacchiarelli et al. [38], we observed no obvious advantage in using miR-1, miR-133a, miR-133b and miR-206, over creatine kinase. Indeed, these miRNAs are enriched in fibers and thought to leak out of damaged myofibers, just as CK does, thus reflecting the same pathological phenomena and providing similar information. Obviously however, the downregulation of miR-31 results from another pathological mechanism, potentially providing complementary information and might give advantage in analyzing circulating miRNA over the traditional creatine kinase test.

In conclusion, this study confirms some previously identified [36], [40] and provides several new serum miRNA biomarkers for the DMD mouse model and patients, as well as for four other disease models. Furthermore, a pathologic model-specific miRNA profile was identified for each studied pathology. Taken together, the circulating miRNA profiling technology was found to be highly efficient for diagnostic purposes in mouse models for striated muscular pathologies.

## Supporting Information

Figure S1 **Blood composition in mouse strains.** All mouse (n = 6−8/strain) were at the ages of 8 to 14 weeks old. P values are shown with *stands for p≤0.05, **stands for p≤0.01, and ***stands for p≤0.001
(TIF)

Table S1 **All miRNAs tested in the screening based on the Exiqon technology.**
(DOCX)

Table S2 **Cq raw data Exiqon screen.**
(XLSX)

Table S3 **Composition DMD cohort.** Shown are the age, type of mutation, ambulatory status, and glucocorticoids treatment.
(DOCX)

## References

[1] Emery AE (2002) The muscular dystrophies. Lancet 359: 687–695. 11879882 10.1016/S0140-6736(02)07815-7

[2] Hermans MC, Pinto YM, Merkies IS, de Die-Smulders CE, Crijns HJ, et al (2010) Hereditary muscular dystrophies and the heart. Neuromuscul Disord 20: 479–492. 20627570 10.1016/j.nmd.2010.04.008

[3] Jacoby D, McKenna WJ (2012) Genetics of inherited cardiomyopathy. Eur Heart J 33: 296–304. 21810862 10.1093/eurheartj/ehr260 PMC3270042

[4] Gasper MC, Gilchrist JM (2005) Creatine kinase: a review of its use in the diagnosis of muscle disease. Med Health R I 88: 398, 400–394. 16363394

[5] Meune C, Wahbi K, Gobeaux C, Duboc D, Pecker F, et al (2010) N-terminal Pro brain natriuretic peptide is a reliable biomarker of reduced myocardial contractility in patients with lamin A/C gene mutations. Int J Cardiol 151: 160–163. 20627339 10.1016/j.ijcard.2010.05.005

[6] Bartel DP (2009) MicroRNAs: target recognition and regulatory functions. Cell 136: 215–233. 19167326 10.1016/j.cell.2009.01.002 PMC3794896

[7] Landgraf P, Rusu M, Sheridan R, Sewer A, Iovino N, et al (2007) A mammalian microRNA expression atlas based on small RNA library sequencing. Cell 129: 1401–1414. 17604727 10.1016/j.cell.2007.04.040 PMC2681231

[8] Liang Y, Ridzon D, Wong L, Chen C (2007) Characterization of microRNA expression profiles in normal human tissues. BMC Genomics 8: 166. 17565689 10.1186/1471-2164-8-166 PMC1904203

[9] Rosenfeld N, Aharonov R, Meiri E, Rosenwald S, Spector Y, et al (2008) MicroRNAs accurately identify cancer tissue origin. Nat Biotechnol 26: 462–469. 18362881 10.1038/nbt1392

[10] Lu J, Getz G, Miska EA, Alvarez-Saavedra E, Lamb J, et al (2005) MicroRNA expression profiles classify human cancers. Nature 435: 834–838. 15944708 10.1038/nature03702

[11] Eisenberg I, Eran A, Nishino I, Moggio M, Lamperti C, et al (2007) Distinctive patterns of microRNA expression in primary muscular disorders. Proc Natl Acad Sci U S A 104: 17016–17021. 17942673 10.1073/pnas.0708115104 PMC2040449

[12] Sylvius N, Bonne G, Straatman K, Reddy T, Gant TW, et al (2011) MicroRNA expression profiling in patients with lamin A/C-associated muscular dystrophy. Faseb J 25: 3966–3978. 21840938 10.1096/fj.11-182915

[13] Sucharov C, Bristow MR, Port JD (2008) miRNA expression in the failing human heart: functional correlates. J Mol Cell Cardiol 45: 185–192. 18582896 10.1016/j.yjmcc.2008.04.014 PMC2561965

[14] McCarthy JJ, Esser KA, Andrade FH (2007) MicroRNA-206 is overexpressed in the diaphragm but not the hindlimb muscle of mdx mouse. Am J Physiol Cell Physiol 293: C451–457. 17459947 10.1152/ajpcell.00077.2007

[15] Lawrie CH, Gal S, Dunlop HM, Pushkaran B, Liggins AP, et al (2008) Detection of elevated levels of tumour-associated microRNAs in serum of patients with diffuse large B-cell lymphoma. Br J Haematol 141: 672–675. 18318758 10.1111/j.1365-2141.2008.07077.x

[16] Chen X, Ba Y, Ma L, Cai X, Yin Y, et al (2008) Characterization of microRNAs in serum: a novel class of biomarkers for diagnosis of cancer and other diseases. Cell Res 18: 997–1006. 18766170 10.1038/cr.2008.282

[17] Mitchell PS, Parkin RK, Kroh EM, Fritz BR, Wyman SK, et al (2008) Circulating microRNAs as stable blood-based markers for cancer detection. Proc Natl Acad Sci U S A 105: 10513–10518. 18663219 10.1073/pnas.0804549105 PMC2492472

[18] Gilad S, Meiri E, Yogev Y, Benjamin S, Lebanony D, et al (2008) Serum microRNAs are promising novel biomarkers. PLoS One 3: e3148. 18773077 10.1371/journal.pone.0003148 PMC2519789

[19] Creemers EE, Tijsen AJ, Pinto YM (2011) Circulating microRNAs: novel biomarkers and extracellular communicators in cardiovascular disease? Circ Res 110: 483–495. 10.1161/CIRCRESAHA.111.247452 22302755

[20] Reid G, Kirschner MB, van Zandwijk N (2011) Circulating microRNAs: Association with disease and potential use as biomarkers. Crit Rev Oncol Hematol 80: 193–208. 21145252 10.1016/j.critrevonc.2010.11.004

[21] Mestdagh P, Van Vlierberghe P, De Weer A, Muth D, Westermann F, et al (2009) A novel and universal method for microRNA RT-qPCR data normalization. Genome Biol 10: R64. 19531210 10.1186/gb-2009-10-6-r64 PMC2718498

[22] Joe H, Ward J (1963) Hierarchical Grouping to Optimize an Objective Function”. Journal of the American Statistical Association. Journal of the American Statistical Association 58: 236–244.

[23] Benjamini Y, Hochberg Y (1995) Controlling the False Discovery Rate: a Practical and Powerful Approach to Multiple Testing. Journal of the Royal Statistical Society 57: 289–300.

[24] Duclos F, Straub V, Moore SA, Venzke DP, Hrstka RF, et al (1998) Progressive muscular dystrophy in alpha-sarcoglycan-deficient mice. J Cell Biol 142: 1461–1471. 9744877 10.1083/jcb.142.6.1461 PMC2141773

[25] Hack AA, Ly CT, Jiang F, Clendenin CJ, Sigrist KS, et al (1998) Gamma-sarcoglycan deficiency leads to muscle membrane defects and apoptosis independent of dystrophin. J Cell Biol 142: 1279–1287. 9732288 10.1083/jcb.142.5.1279 PMC2149352

[26] Chapman VM, Miller DR, Armstrong D, Caskey CT (1989) Recovery of induced mutations for X chromosome-linked muscular dystrophy in mice. Proc Natl Acad Sci U S A 86: 1292–1296. 2919177 10.1073/pnas.86.4.1292 PMC286674

[27] Ozawa E, Mizuno Y, Hagiwara Y, Sasaoka T, Yoshida M (2005) Molecular and cell biology of the sarcoglycan complex. Muscle Nerve 32: 563–576. 15937871 10.1002/mus.20349

[28] Sveen ML, Thune JJ, Kober L, Vissing J (2008) Cardiac involvement in patients with limb-girdle muscular dystrophy type 2 and Becker muscular dystrophy. Arch Neurol 65: 1196–1201. 18779423 10.1001/archneur.65.9.1196

[29] Lancioni A, Rotundo IL, Kobayashi YM, D’Orsi L, Aurino S, et al (2011) Combined deficiency of alpha and epsilon sarcoglycan disrupts the cardiac dystrophin complex. Hum Mol Genet 20: 4644–4654. 21890494 10.1093/hmg/ddr398 PMC3209833

[30] Towbin JA (1998) The role of cytoskeletal proteins in cardiomyopathies. Curr Opin Cell Biol 10: 131–139. 9484605 10.1016/s0955-0674(98)80096-3

[31] Arimura T, Helbling-Leclerc A, Massart C, Varnous S, Niel F, et al (2005) Mouse model carrying H222P-Lmna mutation develops muscular dystrophy and dilated cardiomyopathy similar to human striated muscle laminopathies. Hum Mol Genet 14: 155–169. 15548545 10.1093/hmg/ddi017

[32] Worman HJ, Bonne G (2007) “Laminopathies”: a wide spectrum of human diseases. Exp Cell Res 313: 2121–2133. 17467691 10.1016/j.yexcr.2007.03.028 PMC2964355

[33] Vignier N, Schlossarek S, Fraysse B, Mearini G, Kramer E, et al (2009) Nonsense-mediated mRNA decay and ubiquitin-proteasome system regulate cardiac myosin-binding protein C mutant levels in cardiomyopathic mice. Circ Res 105: 239–248. 19590044 10.1161/CIRCRESAHA.109.201251

[34] Olivotto I, Girolami F, Sciagra R, Ackerman MJ, Sotgia B, et al (2011) Microvascular function is selectively impaired in patients with hypertrophic cardiomyopathy and sarcomere myofilament gene mutations. J Am Coll Cardiol 58: 839–848. 21835320 10.1016/j.jacc.2011.05.018

[35] Fougerousse F, Delezoide AL, Fiszman MY, Schwartz K, Beckmann JS, et al (1998) Cardiac myosin binding protein C gene is specifically expressed in heart during murine and human development. Circ Res 82: 130–133. 9440712 10.1161/01.res.82.1.130

[36] Cacchiarelli D, Legnini I, Martone J, Cazzella V, D’Amico A, et al (2011) miRNAs as serum biomarkers for Duchenne muscular dystrophy. EMBO Mol Med 3: 258–265. 21425469 10.1002/emmm.201100133 PMC3112257

[37] Gagan J, Dey BK, Layer R, Yan Z, Dutta A (2011) MicroRNA-378 targets the myogenic repressor MyoR during myoblast differentiation. J Biol Chem 286: 19431–19438. 21471220 10.1074/jbc.M111.219006 PMC3103322

[38] Cacchiarelli D, Incitti T, Martone J, Cesana M, Cazzella V, et al (2011) miR-31 modulates dystrophin expression: new implications for Duchenne muscular dystrophy therapy. EMBO Rep 12: 136–141. 21212803 10.1038/embor.2010.208 PMC3049433

[39] Pritchard CC, Kroh E, Wood B, Arroyo JD, Dougherty KJ, et al (2012) Blood cell origin of circulating microRNAs: a cautionary note for cancer biomarker studies. Cancer Prev Res (Phila) 5: 492–497. 22158052 10.1158/1940-6207.CAPR-11-0370 PMC4186243

[40] Mizuno H, Nakamura A, Aoki Y, Ito N, Kishi S, et al (2011) Identification of muscle-specific microRNAs in serum of muscular dystrophy animal models: promising novel blood-based markers for muscular dystrophy. PLoS One 6: e18388. 21479190 10.1371/journal.pone.0018388 PMC3068182

[41] Greco S, De Simone M, Colussi C, Zaccagnini G, Fasanaro P, et al (2009) Common micro-RNA signature in skeletal muscle damage and regeneration induced by Duchenne muscular dystrophy and acute ischemia. Faseb J 23: 3335–3346. 19528256 10.1096/fj.08-128579

[42] Townley-Tilson WH, Callis TE, Wang D (2010) MicroRNAs 1, 133, and 206: critical factors of skeletal and cardiac muscle development, function, and disease. Int J Biochem Cell Biol 42: 1252–1255. 20619221 10.1016/j.biocel.2009.03.002 PMC2904322

[43] Cacchiarelli D, Martone J, Girardi E, Cesana M, Incitti T, et al (2010) MicroRNAs involved in molecular circuitries relevant for the Duchenne muscular dystrophy pathogenesis are controlled by the dystrophin/nNOS pathway. Cell Metab 12: 341–351. 20727829 10.1016/j.cmet.2010.07.008

[44] Kosaka N, Iguchi H, Yoshioka Y, Takeshita F, Matsuki Y, et al (2010) Secretory mechanisms and intercellular transfer of microRNAs in living cells. J Biol Chem 285: 17442–17452. 20353945 10.1074/jbc.M110.107821 PMC2878508

[45] Muchir A, Pavlidis P, Decostre V, Herron AJ, Arimura T, et al (2007) Activation of MAPK pathways links LMNA mutations to cardiomyopathy in Emery-Dreifuss muscular dystrophy. J Clin Invest 117: 1282–1293. 17446932 10.1172/JCI29042 PMC1849984

[46] Fichtlscherer S, Zeiher AM, Dimmeler S (2011) Circulating microRNAs: biomarkers or mediators of cardiovascular diseases? Arterioscler Thromb Vasc Biol 31: 2383–2390. 22011751 10.1161/ATVBAHA.111.226696

