# Supplementary figures and images for: Distinctive Serum miRNA Profile in Mouse Models of Striated Muscular Pathologies
Source: PLoS One. 2013 Feb 13;8(2):e55281. doi: 10.1371/journal.pone.0055281 (PMC3572119; doi:10.1371/journal.pone.0055281)

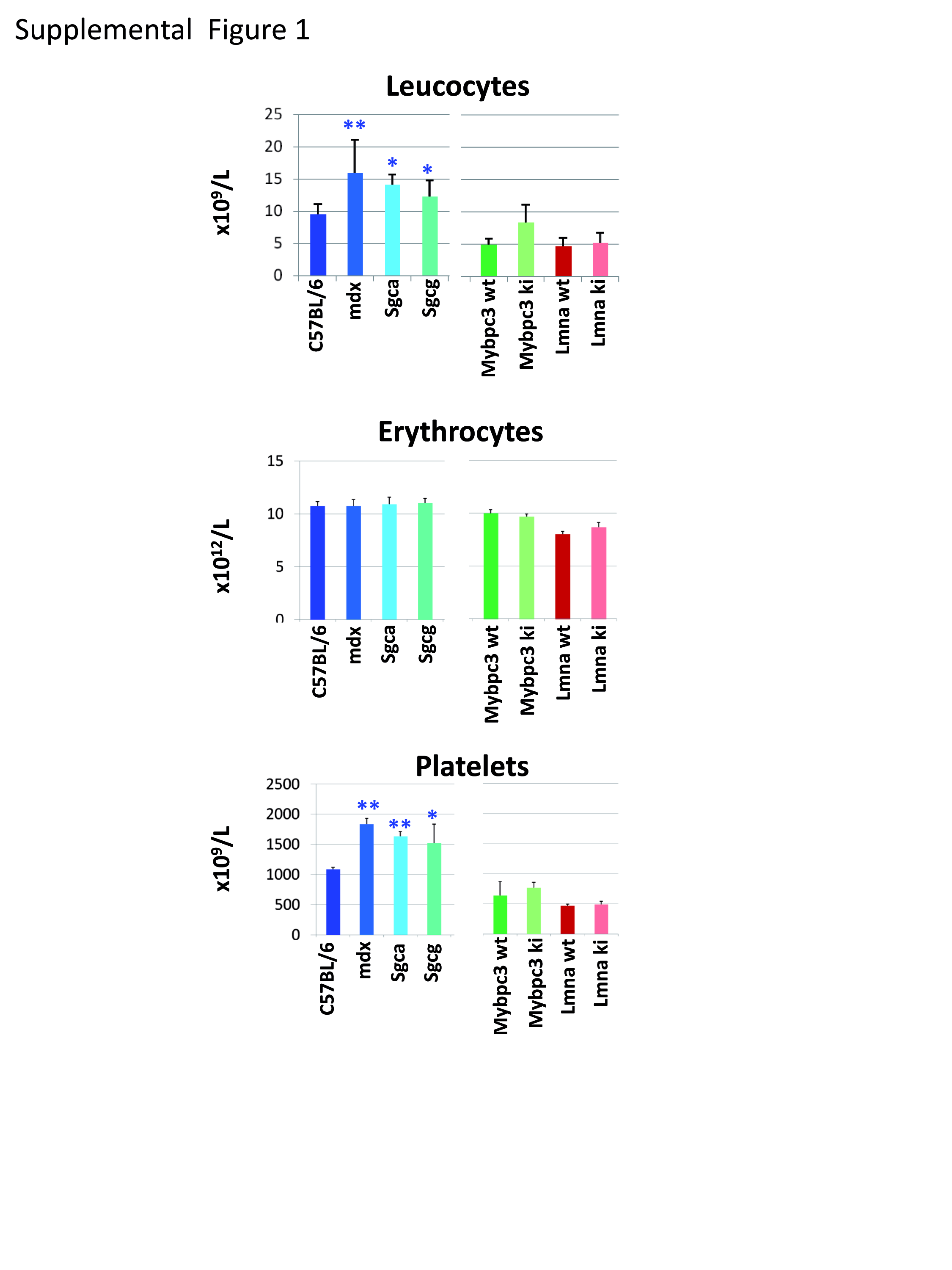

Supplement: Figure S1 — Blood composition in mouse strains. All mouse (n = 6−8/strain) were at the ages of 8 to 14 weeks old. P values are shown with *stands for p≤0.05, **stands for p≤0.01, and ***stands for p≤0.001 (TIF) [file pone.0055281.s001.tif]
