# Supplementary material for: Distinctive Serum miRNA Profile in Mouse Models of Striated Muscular Pathologies
Source: PLoS One. 2013 Feb 13;8(2):e55281. doi: 10.1371/journal.pone.0055281 (PMC3572119; doi:10.1371/journal.pone.0055281)
Supplement: Table S1 — All miRNAs tested in the screening based on the Exiqon technology. (DOCX) [file pone.0055281.s002.docx]

Vignier et al. Supplemental table 1

| Supplemental Table 1 : miRNA analysed in Exiqon screen | | | | |
| --- | --- | --- | --- | --- |
| 1 | mmu-let-7b |  | 44 | mmu-miR-22 |
| 2 | mmu-let-7g |  | 45 | mmu-miR-223 |
| 3 | mmu-let-7i |  | 46 | mmu-miR-23a |
| 4 | mmu-miR-1 |  | 47 | mmu-miR-26a |
| 5 | mmu-miR-101b |  | 48 | mmu-miR-26b |
| 6 | mmu-miR-106a |  | 49 | mmu-miR-27a |
| 7 | mmu-miR-106b |  | 50 | mmu-miR-28* |
| 8 | mmu-miR-122 |  | 51 | mmu-miR-29a |
| 9 | mmu-miR-125a-5p |  | 52 | mmu-miR-29b |
| 10 | mmu-miR-125b-5p |  | 53 | mmu-miR-29c |
| 11 | mmu-miR-130a |  | 54 | mmu-miR-301a |
| 12 | mmu-miR-133a |  | 55 | mmu-miR-301b |
| 13 | mmu-miR-133b |  | 56 | mmu-miR-30a |
| 14 | mmu-miR-135a* |  | 57 | mmu-miR-30a* |
| 15 | mmu-miR-138 |  | 58 | mmu-miR-30b |
| 16 | mmu-miR-142-3p |  | 59 | mmu-miR-30c |
| 17 | mmu-miR-143 |  | 60 | mmu-miR-30d |
| 18 | mmu-miR-145 |  | 61 | mmu-miR-30e |
| 19 | mmu-miR-146a |  | 62 | mmu-miR-30e* |
| 20 | mmu-miR-146b |  | 63 | mmu-miR-31 |
| 21 | mmu-miR-148a |  | 64 | mmu-miR-31* |
| 22 | mmu-miR-149 |  | 65 | mmu-miR-320 |
| 23 | mmu-miR-151-3p |  | 66 | mmu-miR-331-3p |
| 24 | mmu-miR-152 |  | 67 | mmu-miR-339-3p |
| 25 | mmu-miR-153 |  | 68 | mmu-miR-34c* |
| 26 | mmu-miR-16 |  | 69 | mmu-miR-374 |
| 27 | mmu-miR-17 |  | 70 | mmu-miR-378 |
| 28 | mmu-miR-185 |  | 71 | mmu-miR-429 |
| 29 | mmu-miR-187 |  | 72 | mmu-miR-433 |
| 30 | mmu-miR-191 |  | 73 | mmu-miR-434-3p |
| 31 | mmu-miR-192 |  | 74 | mmu-miR-451 |
| 32 | mmu-miR-193b |  | 75 | mmu-miR-466d-3p |
| 33 | mmu-miR-195 |  | 76 | mmu-miR-494 |
| 34 | mmu-miR-199a-3p |  | 77 | mmu-miR-500 |
| 35 | mmu-miR-19a |  | 78 | mmu-miR-532-3p |
| 36 | mmu-miR-19b |  | 79 | mmu-miR-539 |
| 37 | mmu-miR-200a |  | 80 | mmu-miR-672 |
| 38 | mmu-miR-200b |  | 81 | mmu-miR-678 |
| 39 | mmu-miR-203 |  | 82 | mmu-miR-687 |
| 40 | mmu-miR-206 |  | 83 | mmu-miR-709 |
| 41 | mmu-miR-20b |  | 84 | mmu-miR-872 |
| 42 | mmu-miR-21 |  | 85 | mmu-miR-877 |
| 43 | mmu-miR-215 |  | 86 | mmu-miR-93* |
|  |  |  | 87 | mmu-miR-96 |
