# Supplementary material for: Distinctive Serum miRNA Profile in Mouse Models of Striated Muscular Pathologies
Source: PLoS One. 2013 Feb 13;8(2):e55281. doi: 10.1371/journal.pone.0055281 (PMC3572119; doi:10.1371/journal.pone.0055281)
Supplement: Table S3 — Composition DMD cohort. Shown are the age, type of mutation, ambulatory status, and glucocorticoids treatment. (DOCX) [file pone.0055281.s004.docx]

| **Age (years)** | **CK** | **Glucocorticoids** | **Mutation** | **Ambulant** |
| --- | --- | --- | --- | --- |
| 6 | 5750 | Yes | Stop codon exon 53 | Yes |
| 5 | 17125 | Yes | Deletion exons 46-51 | Yes |
| 5 | 17850 | Yes | Deletion exons 2-15 | Yes |
| 7 | 19650 | Yes | Deletion exon 29 | Yes |
| 7 | 7125 | Yes | Deletion exons 48-52 | Yes |
